# Evaluation of Hydrogen Peroxide as an Environmental Model of Parkinson’s Disease in *Drosophila* Larvae

**DOI:** 10.17912/micropub.biology.002344

**Published:** 2026-10-01

**Authors:** Sarah Perry, Frankie Davis, Sydney Cardenas, Vishva Patel

**Affiliations:** 1 Biology, Austin Peay State University, Clarksville, TN, United States

## Abstract

Parkinson’s disease (PD) is characterized by motor dysfunction and dopaminergic neuron loss and is influenced by genetic and environmental factors. Here, we evaluated hydrogen peroxide (H₂O₂) as an accessible oxidative stress model of PD in *Drosophila* larvae. High H₂O₂ concentrations caused lethality or severe developmental impairment, whereas animals exposed to 0.005–0.025% H₂O₂ progressed through larval development and to pupation. Pupation timing was similar to untreated controls at 0.005% and 0.01% H₂O₂ but was delayed by 0.025% H₂O₂. Low-dose H₂O₂ reduced crawling speed and impaired navigational performance but did not cause detectable dopaminergic neuron loss or exacerbate locomotor deficits associated with A53T α-synuclein expression. Thus, H₂O₂ produces behavioral effects without fully recapitulating PD pathology and provides an accessible model for investigating developmental oxidative stress and its potential long-term consequences.

**Figure 1. Hydrogen peroxide exposure produces locomotor deficits without dopaminergic neuron loss in Drosophila larvae:** A) Survivorship and developmental timing qualitative observations for wandering larvae in various concentrations of H _2_ O _2_ . B) Pupation timing for animals reared on various concentrations of H _2_ O _2_ . Comparisons between treated and control groups were made with a Mann-Whitney U test (N = 7) C) Crawling speed data for untreated and H _2_ O _2_ -treated *w ^1118^* larvae. Treated and untreated groups were compared using a Mann-Whitney U test (N = 11-21). D) Schematic of the Ring maze assay. E) Ring maze performance data for untreated and H _2_ O _2_ -treated *w ^1118^* larvae. Treated and untreated groups were compared using a Kolmogorov-Smirnov test (N = 39-47). F) Representative anti-TH confocal images of the larval CNS for untreated and 0.025% H _2_ O _2_ treated animals. G) DAN (TH-positive) cell counts for untreated and 0.025% H _2_ O _2_ treated animals in each of four major clusters and in total. Untreated and treated groups were compared using a Mann-Whitney U test (N = 14-16). H-I) Ring maze performance of TH-Gal4/+ control larvae and TH > aSyn.A53T larvae untreated and treated with H _2_ O _2_ . Statistical comparisons were made using a Kolmogorov-Smirnov test (N = 27-50). ***p < 0.001, **p < 0.01, *p < 0.05, (ns) p > 0.05

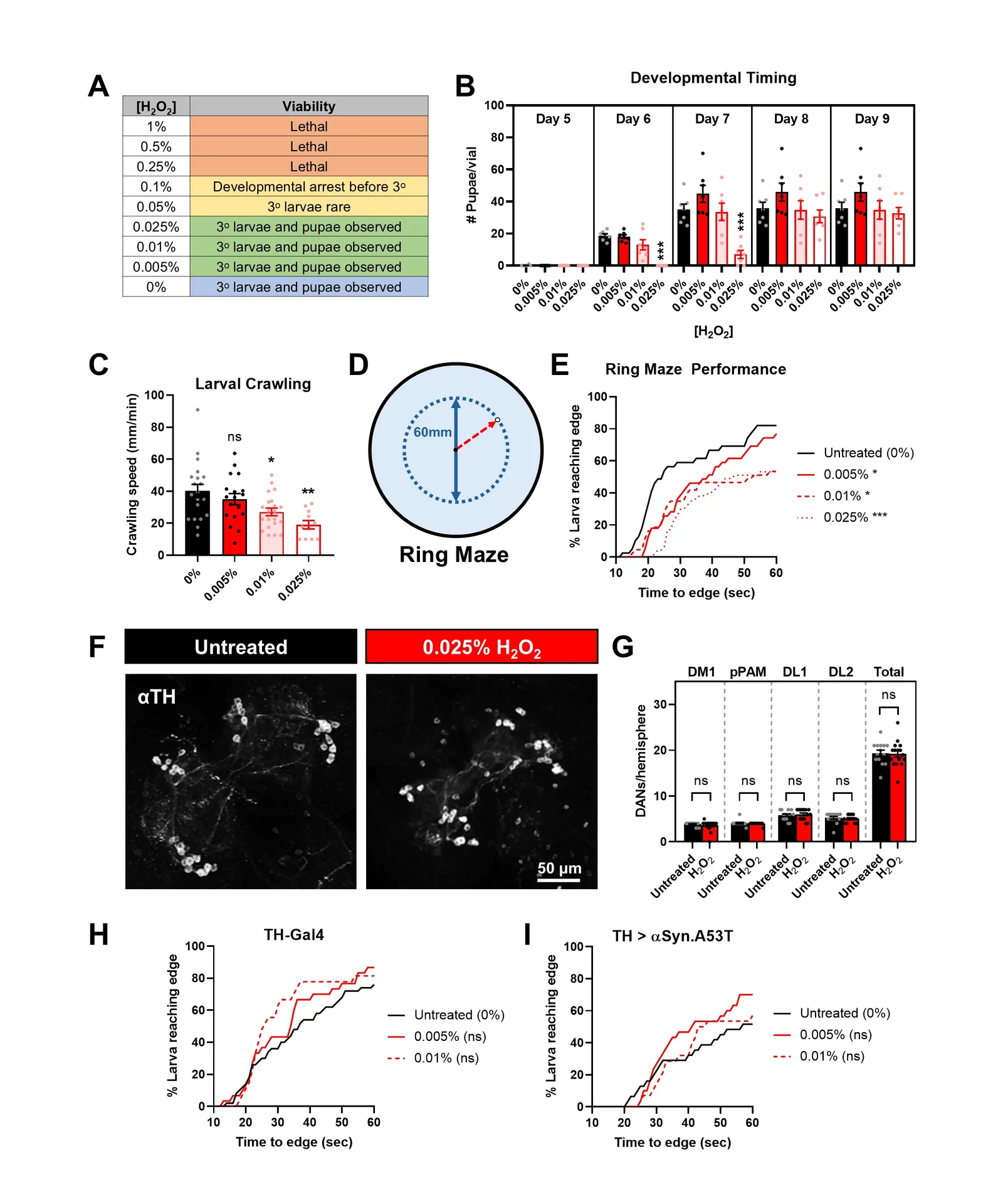



## Description

Parkinson’s disease (PD) is the second most common neurodegenerative disease and is characterized by progressive motor dysfunction associated with the loss of dopaminergic neurons in the substantia nigra pars compacta (Bové et al., 2005). The development of PD is influenced by a complex combination of genetic and environmental factors, making model organisms essential for investigating disease mechanisms and gene–environment interactions (Bové et al., 2005; Varga et al., 2014). *Drosophila* *melanogaster* has become a valuable model for studying PD, and recent work has demonstrated that *Drosophila* larvae provide a particularly simple and accessible system for examining PD-associated locomotor and neurodegenerative phenotypes (Blosser et al., 2020; Perry et al., 2026; Perry & More, 2025; Varga et al., 2014).

Environmental models of PD frequently rely on neurotoxins, including 6-hydroxydopamine (6-OHDA), MPTP, rotenone, and paraquat (Bové et al., 2005). In *Drosophila* larvae, rotenone exposure produces several cardinal PD-associated phenotypes, including locomotor deficits and dopaminergic neuron loss, and can interact with genetic models involving α-synuclein misexpression (Varga et al., 2014). Although these neurotoxins provide powerful tools for modeling environmentally induced PD, their toxicity can limit their suitability for student-centered laboratories and course-based undergraduate research experiences. Hydrogen peroxide (H₂O₂), which can elevate oxidative stress when administered orally to *Drosophila* (Vrailas-Mortimer et al., 2012), may provide a more accessible alternative for investigating oxidative stress as an environmental contributor to PD-like phenotypes.

In this study, we evaluated H₂O₂ exposure as a potential larval *Drosophila* model of environmentally induced PD. We examined the effects of increasing H₂O₂ concentrations on development, crawling speed, navigational efficiency, and dopaminergic neuron number. We further investigated whether H₂O₂ exposure interacts with α-synuclein misexpression to exacerbate locomotor dysfunction. Together, these experiments assess the utility of H₂O₂ exposure as a simple model for investigating environmental and genetic contributions to PD-associated phenotypes.

Results:

We first sought to identify concentrations of H₂O₂ that could produce measurable phenotypes while permitting development. Various quantities of 30% H₂O₂ were added to 5 mL of standard cornmeal food (NutriFly, Bloomington formulation) to generate final concentrations ranging from 0.005% to 1%. Concentrations of 0.25% and higher were lethal, while 0.1% H₂O₂ resulted in developmental arrest before the third-instar stage and third-instar larvae were rarely observed at 0.05%. In contrast, third-instar larvae and pupae were observed following exposure to 0.005–0.025% H₂O₂ ( Figure 1A ). We therefore quantitatively examined developmental timing at these lower concentrations by monitoring pupation. Exposure to 0.005% or 0.01% H₂O₂ did not substantially alter the timing of pupation compared with untreated controls, whereas 0.025% H₂O₂ delayed pupation ( Figure 1B ). Thus, low concentrations of H₂O₂ are compatible with continued development, although developmental delay emerges at the upper end of this concentration range. Based on these observations, we selected 0.005%, 0.01%, and 0.025% H₂O₂ for subsequent experiments.

Motor dysfunction is a hallmark of PD and can be modeled in *Drosophila* larvae through both genetic manipulations, including α-synuclein misexpression, and exposure to neurotoxins such as rotenone (Varga et al., 2014). To determine whether H₂O₂ exposure similarly disrupts locomotion, larvae were raised on food containing 0.005%, 0.01%, or 0.025% H₂O₂, and crawling speed was measured using a standard gridline assay. Exposure to 0.01% and 0.025% H₂O₂ resulted in a significant reduction in crawling speed compared with untreated controls, while 0.005% H₂O₂ did not significantly alter crawling speed ( Figure 1C ).

PD models in *Drosophila* larvae also exhibit impaired navigational efficiency, characterized by increased turning and a reduced ability to maintain directed movement (Blosser et al., 2020; Perry et al., 2026; Perry & More, 2025). We assessed navigational performance using the Ring Maze assay, which measures the ability of an individual larva to travel from the center of an agarose arena to the edge of a 60-mm ring ( Figure 1D ). Larvae exposed to all three concentrations of H₂O₂ exhibited poorer Ring Maze performance compared with untreated controls ( Figure 1E ). Together, these results demonstrate that low-dose H₂O₂ exposure is sufficient to produce motor and navigational deficits in *Drosophila* larvae.

A second hallmark of PD is the progressive loss of dopaminergic neurons (DANs), a phenotype that can also be observed in larval *Drosophila* models following α-synuclein misexpression or rotenone exposure (Varga et al., 2014). To determine whether H₂O₂ exposure similarly affects DAN survival, we used anti-tyrosine hydroxylase (TH) immunohistochemistry and confocal microscopy to quantify DANs within the four major clusters of the anterior larval brain. DAN number was compared between untreated larvae and larvae exposed to 0.025% H₂O₂. We observed no significant difference in the number of DANs within any individual cluster in the anterior brain (DM1, pPAM, DL1, DL2) or in the total number of DANs per brain hemisphere ( Figure 1F –G). Thus, although H₂O₂ exposure produced locomotor and navigational deficits, these behavioral phenotypes were not accompanied by detectable dopaminergic neuron loss.

Finally, we investigated whether H₂O₂ exposure could enhance the effects of a genetic PD model by exposing larvae expressing the A53T mutant variant of α-synuclein in dopaminergic neurons (TH-Gal4 > αSyn.A53T) to low concentrations of H₂O₂. We first examined the effects of H₂O₂ on the TH-Gal4/+ genetic control. Unlike the genetic background used in our initial experiments ( *w ^1118^* ), TH-Gal4 larvae did not exhibit significant Ring Maze deficits following exposure to the two lowest H₂O₂ concentrations ( Figure 1H ). We next examined H₂O₂ exposure in TH-Gal4 > αSyn.A53T larvae. H₂O₂ treatment did not significantly worsen Ring Maze performance compared with untreated A53T-expressing larvae ( Figure 1I ). Therefore, under the conditions tested, H₂O₂ exposure did not demonstrate a clear interaction with A53T α-synuclein misexpression.

Discussion:

In this study, we evaluated oral H₂O₂ exposure as a potential alternative to traditional neurotoxins for modeling environmentally induced PD-like phenotypes in *Drosophila* larvae. We identified a relatively narrow range of H₂O₂ concentrations that permitted continued development while producing measurable behavioral effects. Concentrations of 0.005–0.025% permitted progression to the wandering third-instar and pupal stages, whereas higher concentrations produced severe developmental impairment or lethality.

The developmental effects of H₂O₂ were strongly dose dependent. Although 0.025% H₂O₂ delayed pupation, animals exposed to 0.005% and 0.01% H₂O₂ exhibited pupation timing similar to untreated controls. Importantly, H₂O₂ exposure impaired navigational performance even at these lower concentrations, suggesting that the observed behavioral effects cannot be attributed solely to generalized developmental delay. Within this low-dose range, H₂O₂ also reduced crawling speed at 0.01% and 0.025%, demonstrating that elevated oxidative stress can produce motor phenotypes resembling those observed in other larval PD models.

However, H₂O₂ exposure did not consistently reproduce other hallmark features of PD. Despite the observed behavioral deficits, treatment with 0.025% H₂O₂ did not result in detectable loss of dopaminergic neurons. Additionally, H₂O₂ exposure did not exacerbate the navigational deficits associated with A53T α-synuclein misexpression. These findings suggest that the locomotor effects of H₂O₂ may reflect broader consequences of oxidative stress rather than a specific degeneration of dopaminergic circuitry. Thus, under the conditions examined here, H₂O₂ does not appear to provide a complete substitute for established neurotoxin models of PD.

An important potential application of this model is the investigation of long-term consequences of developmental oxidative stress. Although H₂O₂ exposure produced locomotor deficits during the larval stage, we did not detect dopaminergic neuron loss at this developmental time point. Importantly, animals exposed to 0.005–0.025% H₂O₂ were capable of progressing to pupation. Because PD is an age-dependent neurodegenerative disorder, the absence of detectable dopaminergic neuron loss during larval development does not exclude the possibility that early oxidative stress could produce persistent or delayed effects on neuronal function or survival. Future studies could therefore examine adult motor function, dopaminergic neuron survival, and other PD-associated phenotypes following developmental H₂O₂ exposure. Such experiments could establish whether oxidative stress experienced during development alters susceptibility to neurodegenerative phenotypes later in life.

Interestingly, sensitivity to H₂O₂ also appeared to vary between genetic backgrounds. While H₂O₂ impaired Ring Maze performance in the initial control strain ( *w ^1118^* ), the TH-Gal4 background appeared relatively insensitive to the lower concentrations tested. This observation highlights genetic background as an important consideration when designing experiments involving oxidative stress and warrants further investigation.

Although H₂O₂ treatment did not consistently replicate PD-associated phenotypes, our results provide a useful characterization of the dose-dependent effects of oral H₂O₂ exposure in *Drosophila* larvae. The concentration range identified here may serve as a practical starting point for future studies seeking to manipulate oxidative stress while maintaining development. These findings may therefore be useful for student-centered and course-based research projects investigating oxidative stress, environmental stressors, genetic differences in stress susceptibility, and the long-term consequences of developmental exposure.

## Methods

**Fly genetics and husbandry:** Flies were reared under standard conditions on cornmeal food (NutriFly, Bloomington formulation) at 25°C in a 12-hour light/dark cycle incubator. Larval density for crosses was controlled by pairing 6–7 females with 3–5 males and allowing the crosses to seed for 2–3 days before transferring the parents to a new vial. Wandering third-instar larvae were used for behavioral and immunohistochemical experiments. *w ^1118^* larvae were used in experiments pertaining to the initial dose-response, developmental, locomotor, and DAN analyses. The TH-Gal4 control genotype was generated by outcrossing TH-Gal4 females to *w ^1118^* males, and the resulting progeny were used for experiments. Fly stocks used in this study (obtained from the Bloomington Drosophila Stock Center, BDSC) were as follows: *w ^1118^* (Perry lab stock), TH-Gal4 (BDSC_8848), and UAS-A53T (BDSC_8148).

**Hydrogen peroxide treatment:** Various volumes of 30% H₂O₂ were pipetted on top of 5 mL of cornmeal food (NutriFly, Bloomington formulation) in a standard narrow vial and allowed to diffuse into the media. For example, to achieve a final concentration of 0.025%, 4.17 μL of 30% H₂O₂ solution was added to 5 mL of food. We have also tried mixing H₂O₂ solution into molten food and dispensing it into vials, which produces similar results. Adults were placed on treated food on the same day as preparation.

**Pupation assay:** Mated *w ^1118^* flies were placed on untreated or H₂O₂-treated food at a density of five females and three males per vial and allowed to lay eggs for 24 h before being removed. All parental flies were collected from the same stock bottle to minimize differences in parental age between treatment groups. The number of pupae in each vial was recorded daily through 9 days after egg laying (AEL), by which time all surviving animals had pupated. Cumulative pupation was used to compare developmental timing among treatment groups.

**Gridline crawling assay:** Wandering third-instar larvae were collected with a damp brush, briefly rinsed in tap water to remove residual food, and individually transferred to a 100-mm plate containing 1% agarose positioned over 5-mm grid paper. The number of grid lines each larva crossed during a 2-min trial period was manually recorded and converted into average crawling speed (mm/min). The timer was started only after the first peristaltic contraction, which was considered the first attempt at forward motion. If a larva reached the edge of the plate before the end of the 2-min trial, the timer was stopped and the elapsed time was recorded.

**Ring Maze assay:** Individual wandering third-instar larvae were collected with a damp brush, rinsed briefly in tap water to remove residual food, and transferred to the center of a fresh 100-mm plate containing 1% agarose positioned over a template containing a 60-mm-diameter ring. Larvae were allowed to acclimate and reorient, and timing began once the larva initiated movement away from the center of the arena. The trial ended when the larva’s mouth hooks reached the edge of the 60-mm ring or after a maximum of 60 s. Larvae reaching the ring within 60 s were scored as successful, and the elapsed time to reach the ring was recorded. Larvae that did not reach the ring within 60 s were scored as failures. A fresh agarose plate was used for each trial. Details of Ring Maze statistical analysis can be found in our previous study (Perry et al., 2026). A detailed, user-friendly protocol is attached as supplement.

**Immunohistochemistry and confocal microscopy:** Wandering third-instar larvae were dissected in cold phosphate-buffered saline (PBS) using a body-wall fillet preparation with the central nervous system (CNS) left attached, allowing control and treated samples to be processed together within the same tube. Preparations were initially fixed while pinned on the dissection plate for 10 min in 4% paraformaldehyde (PFA) in PBST (PBS containing 1% Triton X-100), transferred to tubes containing 4% PFA on ice, and subsequently fixed for an additional 30 min with agitation at room temperature. Samples were washed three times for 10 min each in PBST and blocked for 30 min in 5% normal donkey serum (NDS) in PBST at room temperature with agitation. Preparations were incubated overnight at room temperature with agitation in primary antibodies diluted in blocking solution: rabbit anti-tyrosine hydroxylase (TH; 1:200; Novus Biologicals, NB300-109) and mouse anti-Bruchpilot (BRP; nc82; 1:100; Developmental Studies Hybridoma Bank [DSHB]). Following three 10-min washes in PBST, samples were incubated for 2 h at room temperature with agitation in goat anti-rabbit Cy3 (1:200; Fisher Scientific, AP132CMI) and goat anti-mouse Alexa Fluor 488 (1:200; Fisher Scientific, A11001) secondary antibodies diluted in blocking solution. Samples were then washed three times for 10 min each in PBST and stored overnight at 4°C in 80% glycerol. Preparations were mounted in VECTASHIELD and imaged on a Nikon Eclipse Ti confocal microscope using a 10× air objective. Images were formatted and analyzed using ImageJ.
